# Tuberculous dilated cardiomyopathy: an under-recognized entity?

**DOI:** 10.1186/1471-2334-5-29

**Published:** 2005-04-27

**Authors:** Ritesh Agarwal, Puneet Malhotra, Anshu Awasthi, Nandita Kakkar, Dheeraj Gupta

**Affiliations:** 1 Department of Pulmonary Medicine, Post-Graduate Institute of Medical Education and Research, Sector-12, Chandigarh-160012, India; 2 Department of Histopathology, Post-Graduate Institute of Medical Education and Research, Sector-12, Chandigarh-160012, India

## Abstract

**Background:**

Tuberculosis (TB) is a common public health problem in many parts of the world. TB is generally believed to spare these four organs-heart, skeletal muscle, thyroid and pancreas. We describe a rare case of myocardial TB diagnosed on a post-mortem cardiac biopsy.

**Case presentation:**

Patient presented with history suggestive of congestive heart failure. We describe the clinical presentation, investigations and outcome of this case, and review the literature on the involvement of myocardium by TB.

**Conclusion:**

Involvement of myocardium by TB is rare. However it should be suspected as a cause of congestive heart failure in any patient with features suggestive of TB. Increasing recognition of the entity and the use of endomyocardial biopsy may help us detect more cases of this "curable" form of cardiomyopathy.

## Background

Tuberculosis (TB) is generally believed to spare these four organs-heart, thyroid, pancreas and skeletal muscle. Involvement of myocardium by TB is rare, and generally occurs in conjunction with pericardial involvement. Isolated myocardial TB is a rare finding, and definitive diagnosis during life requires a myocardial biopsy. Herein we describe a patient who presented with features suggestive of congestive heart failure, and was finally diagnosed to have myocardial TB on a post-mortem cardiac biopsy.

## Case presentation

**Figure 1:**
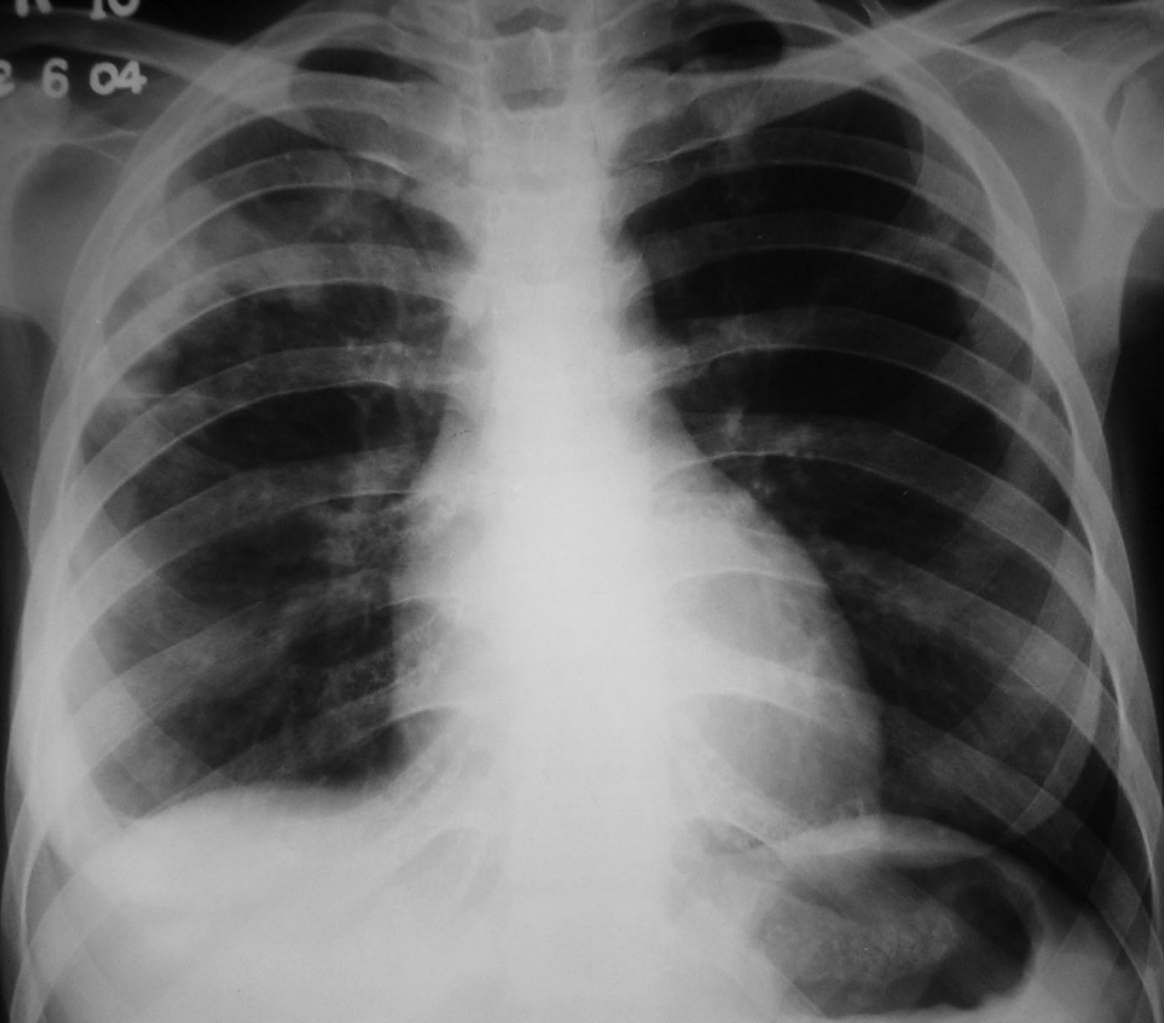
Chest radiograph showing right upper lobe consolidation (cardiac silhouette is normal).

**Figure 2:**
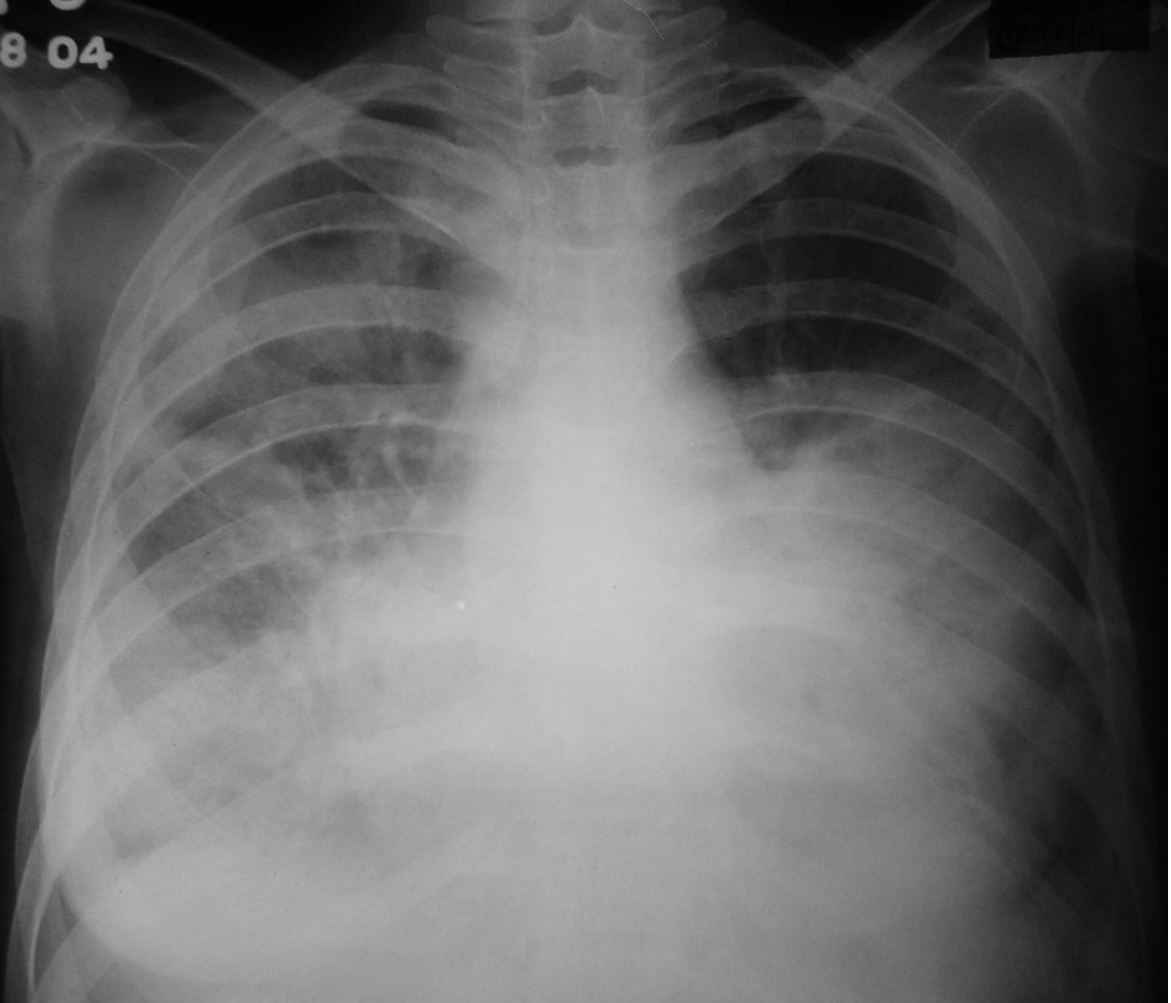
Chest radiograph bilateral alveolar opacities and cardiomegaly.

**Figure 3:**
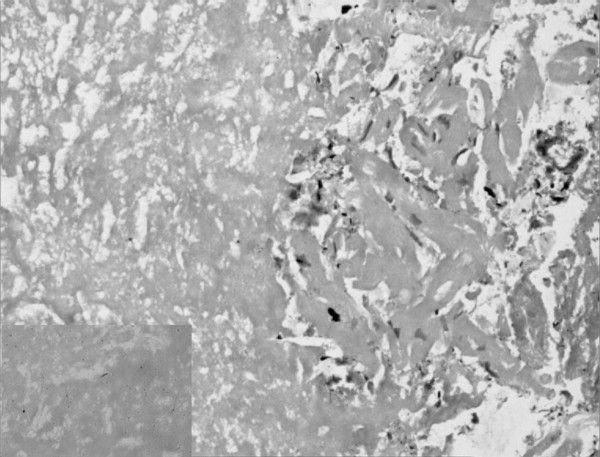
Photomicrograph demonstrates large areas of necrosis with interspersed myocytes showing degeneration and regeneration [Hematoxylin & Eosin, original magnification × 33]. Inset shows photomicrograph demonstrating acid-fast bacilli [Ziehl-Neelson stain, original magnification × 33].

A 25-year old female presented to the emergency department with a one week history of low grade fever, increasing cough and dyspnea on exertion. At presentation she had dyspnea at rest, orthopnea and paroxysmal nocturnal dyspnea. Around two and a half months before the present illness, she had low grade fever, anorexia and weight loss, cough with expectoration and hemoptysis. She was investigated at another center and a sputum smear for acid-fast bacilli was positive. She was started on antituberculous therapy with which she improved symptomatically. She experienced weight gain, and her appetite became normal. A sputum smear performed after two months for acid-fast bacilli was negative. On examination, the patient was conscious and afebrile with a pulse rate of 128 beats/minute, blood pressure of 100/60 mm Hg and a respiratory rate of 38/minute. She had bilateral pitting pedal edema. Examination of the cardiovascular system revealed tachycardia, elevated jugular venous pressure, diffuse apical impulse and left ventricular third heart sound. Auscultation of the chest showed bibasal inspiratory crackles. She also had tender hepatomegaly and free fluid in the abdomen. Her oxygen saturation was 85% on pulse oximetry. Arterial blood gases showed type 1 respiratory failure [(FiO2 0.5) – pH 7.48, PaO2 8.2 kPa, PaCO2 3.6 kPa, HCO3 21 mEq/L]. She was started on supplemental oxygen, intravenous morphine, nitroglycerin, furosemide and dobutamine, oral captopril, stress ulcer and deep venous thrombosis prophylaxis. Chest X-ray (Figure 1) done two months ago showed right upper lobe consolidation with normal cardiac silhouette. A chest radiograph (Figure 2) performed at admission revealed cardiomegaly and bilateral alveolar opacities. Electrocardiogram revealed sinus tachycardia and non-specific ST-T changes in the lateral leads. Echocardiography was performed which revealed global hypokinesia, enlarged left atrium and left ventricle, mild mitral regurgitation, severe left ventricular systolic dysfunction with an ejection fraction of 20%; left ventricular end systolic and end diastolic dimensions were 45 and 51 mm respectively. Right atrium and right ventricle were dilated with associated mild tricuspid regurgitation and pulmonary artery systolic pressure of 47 mm Hg. A provisional diagnosis of viral myocarditis was made and she was shifted to respiratory intensive care unit where noninvasive ventilation (NIV) was initiated with continuous positive airway pressure at 10 cm H_2_O. Arterial line was placed through the right radial artery and continuous electrocardiographic monitoring was performed. As the patient had no improvement with NIV and was becoming hypoxemic and agitated, she was given intravenous fentanyl and oral endotracheal intubation was performed. She was mechanically ventilated with assist/control mode at tidal volumes of 350 mL, rate of 20, PEEP of 10 cm H_2_O, and FiO_2 _of 1. Her peak and plateau pressures were 35 and 28 cm H_2_O respectively. She was sedated intermittently with intravenous fentanyl. Blood cultures, mycoplasma and legionella serology were sent and patient was also started on intravenous azithromycin. Other treatment measures and antituberculous therapy were continued. Biochemical investigations revealed hypoalbuminemia and prerenal azotemia; liver function tests, complete blood, coagulation profile and urinalysis were normal. With mechanical ventilation, oxygen saturation improved and oxygen requirements decreased to 0.4. She had recurrent episodes of nonsustained ventricular tachycardia and amiodarone infusion was started. However on the second day of admission she had a sudden episode of ventricular fibrillation and despite all resuscitative measures the patient could not be revived. Her husband did not give consent for an autopsy; however he agreed for a post-mortem cardiac biopsy, which revealed multi-focal areas of caseous myocardial necrosis, Ziehl-Neelson stain for acid-fast bacilli was positive (Figure 3). HIV serology received postmortem was nonreactive.

## Conclusion

Involvement of heart in tuberculosis (TB) occurs in one to two percent of patients with tuberculosis [1,2]. The most common site involved is pericardium, and tuberculous involvement of the myocardium is exceedingly rare. The introduction of effective antituberculous therapy has further decreased the incidence [3]. The earliest report of myocardial TB was in 1664 by Maurocordat and second report in 1761 by Morgagni [4]. The myocardium can be affected either by direct extension or by retrograde lymphatic drainage from mediastinal nodes; direct spread from tuberculous pericarditis can also occur [5]. Moreover, during the hematogenous phase of dissemination of primary TB, any and every tissue and organ in the body is liable to seeding by mycobacteria and consequent pathological changes.

Myocardial TB is often not diagnosed during life, but if suspected the diagnosis can be established by an endomyocardial biopsy [6]. Three types of myocardial involvement have been described viz. tuberculomas of the myocardium with central caseation (seen in our patient), miliary tubercles of the myocardium complicating generalized miliary disease and the uncommon diffuse infiltrative type associated with tuberculous pericarditis [7]. The right heart, particularly the right atrium, is most often affected, probably because of the frequent involvement of the right mediastinal lymph nodes with consequent involvement of the myocardium [8], although right ventricle [3] and left ventricle [4] have been found to be involved most frequently in different series. Myocardial TB can manifest in various forms. Rhythm disturbances include supraventricular arrhythmias [3,6], ventricular arrhythmias [9] or varying degrees of conduction blocks [10], and sudden cardiac death is also described [5,11]. Right ventricular outflow tract obstruction [3,12,13], ventricular aneurysm [4,13], ventricular pseudoaneurysm [14], aortic insufficiency [15], coronary arteritis [10,13], or congestive heart failure [6,16,17] have also been described in literature. Recently, magnetic resonance imaging has also been used in the diagnosis of myocardial TB [18].

Antitubercular drugs are the cornerstone of therapy [6], and surgery [12,14-16] is indicated only in complicated cases. Paradoxically, our patient presented with features of congestive heart failure, and echocardiography showed severe left ventricular systolic dysfunction inspite of being on anti-tuberculous therapy which had resulted in bacteriological improvement of the pulmonary lesions. The reason for this is not clear, but one likely reason is probably enhanced immunogenicity of the host to tubercle bacilli. This has been called as a 'paradoxical response' [19]. In this regard, at least theoretically, glucocorticoids may have a role along with antituberculous therapy.

In conclusion, although myocardial involvement by tuberculosis is rare, it should be suspected as a cause of congestive heart failure in any patient with features suggestive of TB, as cases of myocardial TB almost always show evidence of TB at other sites [6]. Increasing recognition of the entity, and the use of endomyocardial biopsy may help us detect more cases of this "curable" form of cardiomyopathy especially in areas of high prevalence of TB. It may not be out of place to state.... Look and ye shall find....

## Competing interests

The author(s) declare that they have no competing interests.

## Authors' Contributions

**RA **was involved in patient care and drafting the manuscript

**PM **was involved in patient care and editing the manuscript

**AA **was involved in histopathological examination and editing the manuscript

**NK **was involved in histopathological examination

**DG **conceived the study and was also involved in patient care

## Pre-publication history

The pre-publication history for this paper can be accessed here:


